# Wearable echocardiography enables real-time perioperative monitoring in a pediatric liver transplant recipient with hepatopulmonary syndrome and pulmonary arteriovenous malformations: a case report

**DOI:** 10.1186/s12887-026-07440-y

**Published:** 2026-08-04

**Authors:** Mao Ye, Pengjie Zhang, Chunling Jiang, Bin Zhou, Xinmei Yang, Haibo Song, Weiyi Zhang

**Affiliations:** 1 Department of Anesthesiology, West China Hospital, Sichuan University, Chengdu, 610041 China; 2 Sichuan Wismed Medical Technology Co. Ltd, Chengdu, China

**Keywords:** Wearable echocardiography, Pediatric liver transplant, Hepatopulmonary syndrome, Pulmonary arteriovenous malformation, Perioperative monitoring

## Abstract

**Background:**

Hepatopulmonary syndrome (HPS) with pulmonary arteriovenous malformations (PAVMs) creates high risks of hemodynamic instability and gas embolism during pediatric liver transplantation. Transesophageal echocardiography is relatively contraindicated due to esophageal varices, while intermittent transthoracic echocardiography lacks the temporal resolution to capture transient events. This is the first report of continuous wearable echocardiography for real-time perioperative monitoring in a pediatric liver transplant recipient with HPS and PAVMs.

**Case presentation:**

A 3-year-old girl with biliary atresia and decompensated cirrhosis underwent deceased donor liver transplantation. Preoperative evaluation confirmed severe HPS with PAVMs. During surgery, fluid management was guided by continuous monitoring using wearable echocardiography to maintain hemodynamic stability. In particular, within 10 s after graft reperfusion, wearable echocardiography revealed numerous bright echogenic foci in all visualized cardiac chambers. This real-time finding provided immediate visual confirmation of gas embolism, which was associated with targeted multidisciplinary intervention. The surgery was successfully completed, and the patient was discharged on postoperative day 24.

**Conclusions:**

This is the first detailed report demonstrating the utility of wearable echocardiography for continuous, real-time cardiac monitoring in a pediatric liver transplant recipient with severe HPS and PAVMs. This technology may facilitate individualized fluid management and enable early warning of catastrophic complications in pediatric liver transplantation when standard echocardiographic modalities are contraindicated or insufficient.

**Supplementary Information:**

The online version contains supplementary material available at https://doi.org/10.1186/s12887-026-07440-y.

## Background

Hepatopulmonary syndrome (HPS) affects approximately 7% of pediatric patients with biopsy-proven cirrhosis [1]. Pathologically, HPS is characterized by diffuse pulmonary vasodilatation and intrapulmonary right-to-left shunting, resulting in refractory hypoxemia and a hyperdynamic circulation. This inherently fragile cardiopulmonary profile renders these patients extremely susceptible to life-threatening hemodynamic upheaval during the liver transplantation [2]. Furthermore, HPS is frequently associated with pulmonary arteriovenous malformations (PAVMs), which disrupt the normal pulmonary capillary filtration barrier. As a result, when air enters the venous circulation during portal vein clamping or graft reperfusion, these malformations provide a direct conduit for gas to bypass the pulmonary capillary bed and enter the left heart and systemic circulation — a phenomenon known as paradoxical systemic gas embolism [3].

Given these risks, perioperative monitoring capable of detecting these rapid, transient, and dangerous hemodynamic shifts remains a major unmet clinical need in this high-risk population. Transesophageal echocardiography (TEE) is relatively contraindicated in these children, owing to fragile esophageal mucosa and a high risk of variceal bleeding during probe placement [4]. Conversely, intermittent transthoracic echocardiography (TTE) lacks the temporal resolution to capture these brief, critical events during portal vein clamping and reperfusion. To our knowledge, this is the first report of continuous wearable echocardiography in a pediatric liver transplant recipient with HPS and PAVMs. Although the same device has been described in a critically ill neonate [5], its application in this specific context is novel, where TEE is relatively contraindicated and intermittent TTE insufficient to capture transient events.

### Case presentation

A 3-year-old girl (95 cm, 15 kg) with biliary atresia and decompensated cirrhosis, who had undergone Kasai portoenterostomy at 2 months of age, was listed for deceased donor liver transplantation. Preoperative evaluation revealed marked digital clubbing and severe hypoxemia (room-air SpO₂ 85%; arterial partial pressure of oxygen [PaO₂] 56.6 mmHg). Preoperative contrast-enhanced TTE with agitated saline showed immediate right heart opacification, followed by dense left heart microbubbles within 2–3 cycles that were traced to the pulmonary veins, consistent with intrapulmonary shunting. Additionally, computed tomography pulmonary angiography revealed pulmonary vascular dilation. Collectively, these clinical and imaging findings confirmed a diagnosis of severe HPS with PAVMs [6].

**Table 1:** Intraoperative hemodynamic and echocardiographic parameters during wearable echocardiography-guided fluid titration with 5% albumin

Time from surgery start (min)	Event	HR (beats·min⁻¹)	BP (mmHg)	FiO₂ (%)	SpO₂ (%)	LVEDD (mm)	LVEF (%)	Norepinephrine (μg·kg⁻¹·min⁻¹)	Epinephrine (μg·kg⁻¹·min⁻¹)	Fluid rate(mL·kg⁻¹·h⁻¹)
0	Baseline (post-induction)	106	85/49	60	92	30	70	0	0	5*→*10
134	5 min before portal vein clamping	104	89/62	60	93	34	67	0	0	10
139	Portal vein clamping	107	87/60	60	92	30	67	0.15	0	10*→*15
145	IVC clamping (partial)	100	86/56	60	90	29	67	0.15	0.03	15*→*20
168	IVC unclamping	102	88/55	60	92	31	67	0.15	0.03	20
200	Portal vein unclamping (Graft reperfusion )	103	92/51	60	94	34	67	0.15	0.03	20*→*15
409	Before abdominal closure	93	90/45	60	93	31	77	0.10	0.03	15

Arrows (*→*) indicate changes in the infusion rate during the corresponding periods

*LVEDD* left ventricular end-diastolic diameter, *LVEF* left ventricular ejection fraction

**Fig. 1:**
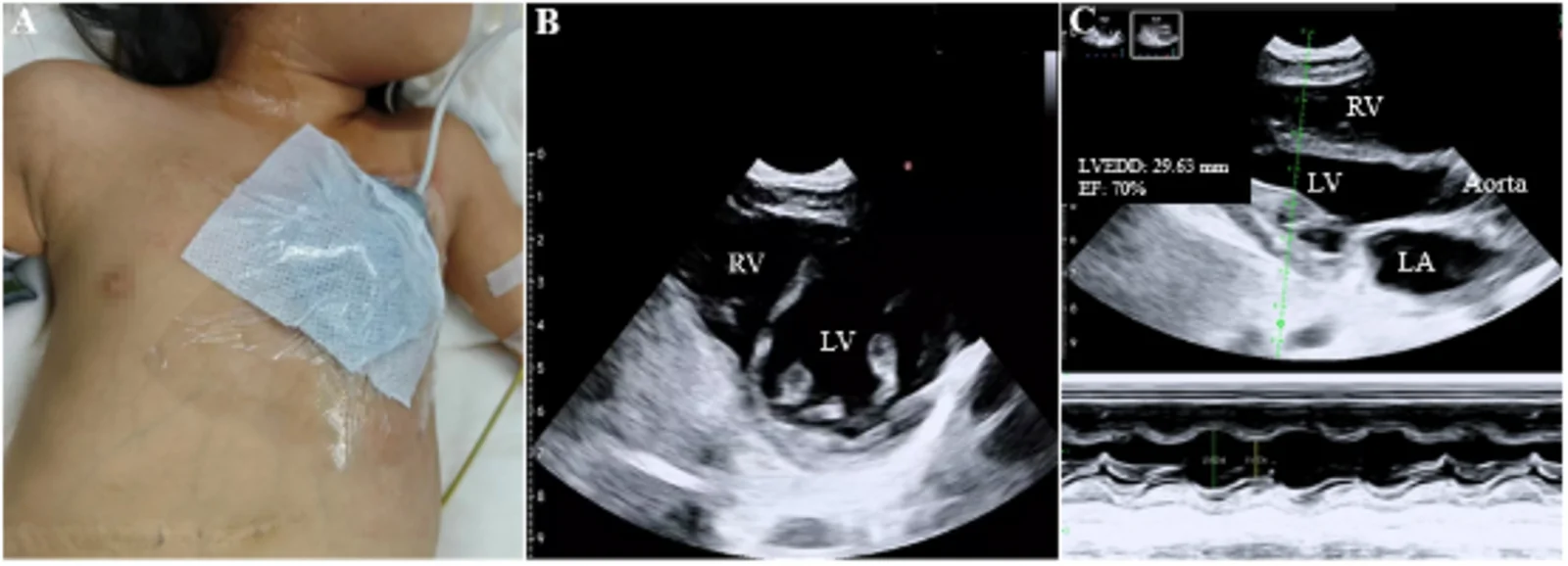
Intraoperative echocardiographic imaging using the wearable device. **A** Clinical setup showing the wearable ultrasound probe placed on the patient’s chest during anesthesia; (**B**) Echocardiographic image of the left ventricle short-axis view showing the right ventricle (RV) and left ventricle (LV); (**C**) Echocardiographic image of the parasternal left ventricle long-axis view showing the RV, LV, left atrium (LA), and aorta

On the day of surgery, a wearable continuous phased-array ultrasound system (Model P2-5-64, center frequency 3.6 MHz; Sichuan Wismed Medical Technology Co., Ltd., Chengdu, Sichuan, China) was applied for intraoperative monitoring. The probe was secured using sterile medical tape and positioned on the left anterior chest wall to obtain a parasternal long-axis view, and maintained stable acoustic contact for the duration of the surgery (Fig. 1). Intraoperative fluid therapy consisted of 5% albumin titrated under real-time guidance using wearable echocardiography (Table 1). Following portal vein clamping, left ventricular end-diastolic diameter (LVEDD) fell from 34 to 30 mm, prompting a fluid increase from 10 to 15 mL·kg⁻¹·h⁻¹. During subsequent partial inferior vena cava (IVC) clamping, LVEDD further decreased to 29 mm, and the rate was increased to 20 mL·kg⁻¹·h⁻¹. After IVC unclamping, LVEDD rose to 31 mm, and the rate was maintained at 20 mL·kg⁻¹·h⁻¹. Upon graft reperfusion, LVEDD increased to 34 mm, guiding a reduction to 15 mL·kg⁻¹·h⁻¹. Before closure, LVEDD remained at 31 mm, with the rate kept at 15 mL·kg⁻¹·h⁻¹. These adjustments reflect the value of continuous wearable echocardiography for real-time, individualized fluid titration.

**Fig. 2:**
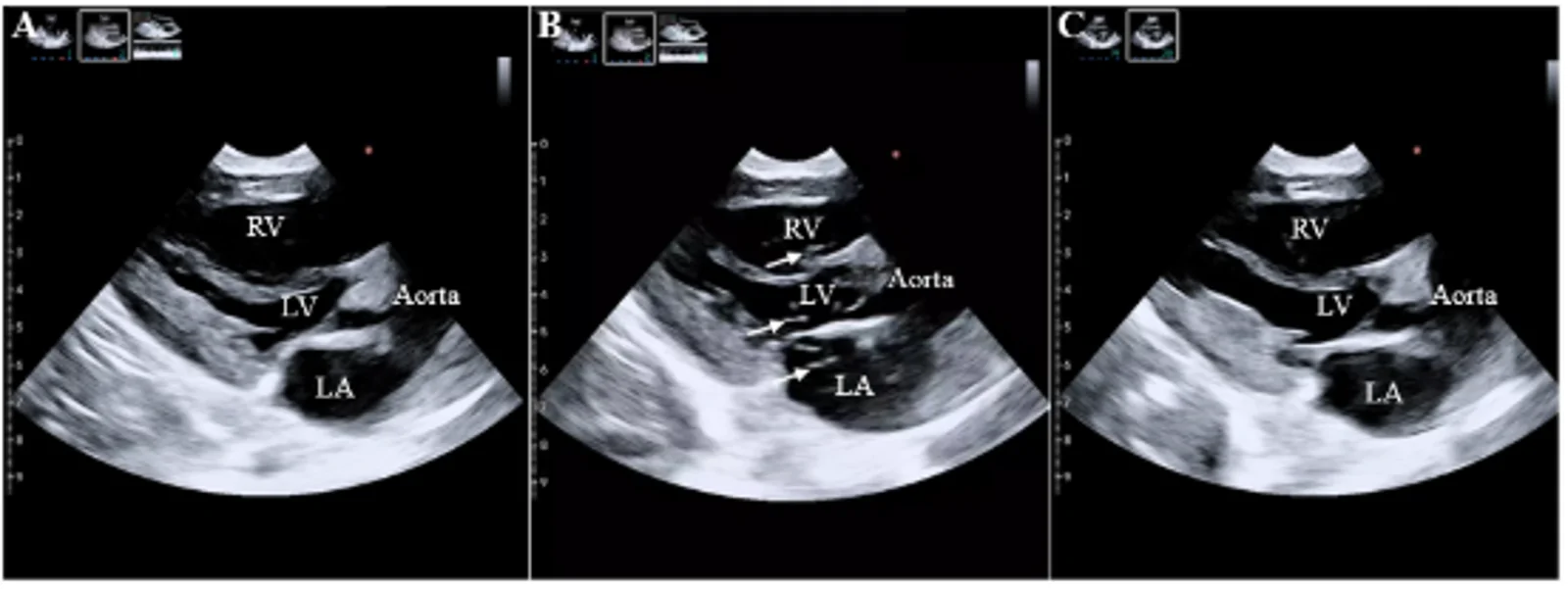
Wearable echocardiography during hepatic portal management. **A** Before reperfusion: no intracardiac gas is seen in the RV, LV, LA, or aorta; (**B**) Immediately after portal vein reperfusion: multiple bright echogenic foci consistent with gas bubbles (indicated by white arrows) are visible in the right ventricle, left ventricle, left atrium, and aorta within the parasternal long-axis view; (**C**) Ten minutes after reperfusion: following multimodal rescue intervention, intracardiac gas has completely resolved

Specifically, graft reperfusion was initiated at 200 min after surgery start (61 min after portal vein clamping). Within 10 s after reperfusion, wearable echocardiography revealed numerous bright echogenic foci within the visualized cardiac chambers (right ventricle, left ventricle, left atrium, and aorta) in the fixed parasternal long-axis view (Fig. 2B). This finding was further supported by concurrent objective measures: end-tidal CO₂ (EtCO₂) dropped abruptly from 35 to 29 mmHg within 15 s, and MAP fell from 70 to 62 mmHg over the same period, consistent with intracardiac gas embolism. This real-time alert prompted immediate multidisciplinary intervention. The surgical team immediately paused further vascular manipulation, systematically inspected potential sites of air entry (including the portal vein anastomosis), and irrigated the surgical field. Although no definitive source of air entry was identified, the echocardiographic findings prompted the team to implement enhanced de-airing precautions during all subsequent anastomoses, including thorough flushing of the graft and venous lines before closure. Meanwhile, the anesthetic team maintained hemodynamic stability. Interventions included 100% oxygen inhalation, Trendelenburg positioning, 5 cm H₂O positive end-expiratory pressure (PEEP), norepinephrine to maintain systemic vascular resistance, and epinephrine to enhance right ventricular function. With multidisciplinary collaboration, intracardiac gas embolism resolved completely within 10 min after reperfusion. A supplementary video shows this process (see Additional file 1). The surgery was successfully completed with a total operative time of 570 min.

Postoperatively, the patient remained intubated and was admitted to the PICU. The postoperative course was complicated by a pulmonary infection requiring antimicrobial therapy and prolonged ventilatory support; the patient was successfully extubated on postoperative day 11. After extubation, supplemental oxygen was administered at 3 L/min via nasal cannula, titrated to SpO₂, and continued until discharge. Neurological examination, including pupillary light reflexes, plantar responses (Babinski sign), cranial nerve function, and motor strength, was performed daily and remained normal throughout the hospital stay. Neuroimaging was not performed in the absence of clinical neurological deficits. The patient was transferred to the general ward on postoperative day 12 and discharged on day 24, with oxygenation stabilized at SpO₂ 85–90% on room air at rest. At the 3-month follow-up, the patient had normal activity level, no neurological sequelae, and mild improvement of finger cyanosis compared with the preoperative status.

## Discussion

This case illustrates the utility of continuous wearable echocardiography for real-time perioperative monitoring in a pediatric liver transplant recipient with severe HPS and PAVMs. The wearable device offered two critical advantages over conventional monitoring modalities in this high-risk population: continuous tracking of preload changes to guide individualized fluid therapy, and real-time detection of transient gas embolism, which was associated with immediate rescue intervention.

In the present case, the diagnosis of severe HPS was confirmed by a preoperative room-air arterial PaO₂ of 56.6 mmHg, which meets the European Respiratory Society (ERS) Task Force criteria for severe HPS (PaO₂ 50–59 mmHg) [6]. Notably, a large longitudinal cohort study demonstrated that in patients with moderate to very severe HPS, PaO₂ declines at a rate of 3.7 mmHg per year [7]. These population-level data underscore the progressive nature of hypoxemia in HPS and highlight the importance of timely liver transplantation, which has been shown to reverse this decline, with PaO₂ increasing by 6.5 mmHg per month in the first year after transplant [8].

The core anesthetic challenge during liver transplantation arises from a pathophysiology-driven volume management dilemma [2]. Portal vein clamping and IVC clamping reduce venous return and preload, while IVC and portal vein unclamping may precipitate acute right ventricular failure due to a sudden surge in venous return combined with ischemia-reperfusion-mediated elevation in pulmonary vascular resistance [9]. These rapid hemodynamic shifts demand real-time monitoring of both preload and right ventricular function, which wearable echocardiography provided in our patient through continuous visualization from a fixed parasternal window. LVEDD was used as a trend indicator for fluid titration, while right ventricular function was assessed qualitatively by evaluating ventricular size, contractility, septal motion, and outflow tract appearance. This integrated information provided a basis for intraoperative fluid management and the use of vasoactive agents.

Additionally, our patient had preoperatively confirmed PAVMs on agitated saline contrast echocardiography. Although microbubbles appeared within 2–3 cardiac cycles, this time window alone cannot reliably distinguish intracardiac from intrapulmonary shunting, particularly in cirrhosis with hyperdynamic circulation where pulmonary vasodilatation accelerates transit time [10, 11]. The diagnosis was therefore based on multiple complementary findings: microbubbles originating from the pulmonary veins, exclusion of intracardiac defects by serial echocardiography, and a clinical presentation consistent with severe HPS. These malformations disrupt the pulmonary capillary filtration barrier, providing a direct conduit for venous air that may be entrained during portal vein clamping or graft reperfusion to bypass the lungs and enter the systemic arterial circulation [12]. This mechanism places patients with PAVMs at risk for paradoxical systemic gas embolism. In a case series of five adult HPS recipients, Chan et al. [13] reported that two patients developed postoperative embolic strokes, with one suffering persistent right hemianopia and the other requiring prolonged hospitalization (84 days) with significant residual deficits. Notably, all events in that series were diagnosed only after neurological symptoms emerged postoperatively, suggesting that intraoperative embolic phenomena may have been missed by conventional intermittent monitoring. In our patient, by contrast, wearable echocardiography identified intracardiac gas embolism within 10 s of portal vein unclamping. We acknowledge that echogenic material upon reperfusion is common, but several features favored gas embolism: the pre-existing right-to-left shunt, exclusion of intracardiac defects, dense left-heart opacification, and concurrent EtCO₂ and MAP drops. This real-time detection prompted immediate multidisciplinary intervention, with complete resolution observed within 10 min, and no postoperative neurological sequelae occurred. This favorable outcome highlights the diagnostic value of real-time echocardiographic confirmation.

While capnography detected the physiological consequences of embolism, it could not distinguish gas embolism from other causes. The wearable device addressed this gap by visually confirming that the echogenic foci were gas bubbles distributed within all visualized cardiac chambers (right ventricle, left ventricle, left atrium, and aorta), thereby converting a nonspecific signal into a specific diagnosis, enabling the team to initiate targeted multidisciplinary intervention. The choice of interventions was based on their physiological rationale and potential trade-offs in the context of intrapulmonary shunting. Low-level PEEP (5 cm H₂O) was administered to maintain oxygenation while minimizing the theoretical risk of exacerbating right-to-left shunting. Regarding positioning, although left lateral decubitus is the standard position for venous air embolism, it was not feasible during liver transplantation; therefore, Trendelenburg positioning was used to reduce the risk of cerebral air embolism. These measures were undertaken based on a comprehensive assessment of their respective benefits and limitations, rather than being regarded as uniformly applicable maneuvers.

The clinical utility of wearable echocardiography in this case was supported by its technical feasibility. The device proved feasible in this 3-year-old child, remaining stable under sterile draping for the entire 9.5-hour procedure without interfering with surgical access. The flexible probe conformed naturally to the body surface and maintained stable acoustic contact throughout the surgery. Our experience aligns with a previous report demonstrating the feasibility of the same device for prolonged monitoring in a critically ill neonate [5], suggesting that wearable echocardiography may serve as a valuable adjunctive monitoring tool in pediatric liver transplantation for children with HPS and PAVMs.

Several limitations should be acknowledged. First, as a single case report, our findings require validation in larger cohorts. Second, the wearable device’s single parasternal window limits comprehensive cardiac assessment, and this limitation is exacerbated during liver transplantation by intraoperative factors such as patient repositioning and surgical manipulation that compromise image quality. Accordingly, LVEDD measurements were used only as dynamic trend indicators rather than absolute quantitative values, and the reliability of quantitative chamber measurements from this single fixed window has not been validated. Third, neurological assessment was incomplete, as cerebral somatic regional oxygenation and postoperative neuroimaging were not performed, though no clinical deficits were observed.

## Conclusions

In conclusion, wearable echocardiography enables real-time, continuous, non-invasive cardiac and hemodynamic monitoring in pediatric liver transplant recipients with severe HPS and PAVMs. It may facilitate individualized fluid management and early warning of gas embolism. This technology represents a promising adjunctive monitoring modality to optimize perioperative anesthetic safety for such high-risk children.

## Supplementary Information

Additional file 1.

Additional file 2.

## Data Availability

No datasets were generated or analysed during the current study.
